# Expression of red/green-cone opsin mutants K82E, P187S, M273K result in unique pathobiological perturbations to cone structure and function

**DOI:** 10.3389/fnins.2024.1368089

**Published:** 2024-02-12

**Authors:** Emily R. Sechrest, Robert J. Barbera, Xiaojie Ma, Frank Dyka, Junyeop Ahn, Brooke A. Brothers, Marion E. Cahill, Isaac Hall, Wolfgang Baehr, Wen-Tao Deng

**Affiliations:** ^1^Department of Ophthalmology and Visual Sciences, West Virginia University, Morgantown, WV, United States; ^2^Department of Ophthalmology, University of Florida, Gainesville, FL, United States; ^3^Department of Chemistry, University of Virginia, Charlottesville, VA, United States; ^4^Department of Biochemistry, West Virginia University, Morgantown, WV, United States; ^5^Department of Biology, West Virginia University, Morgantown, WV, United States; ^6^Department of Natural Sciences, Fairmont State University, Fairmont, WV, United States; ^7^Department of Ophthalmology, John A. Moran Eye Center, University of Utah Health Science Center, Salt Lake City, UT, United States; ^8^Department of Neurobiology and Anatomy, University of Utah Health Science Center, Salt Lake City, UT, United States; ^9^Department of Biology, University of Utah, Salt Lake City, UT, United States

**Keywords:** blue cone monochromacy (BCM), cone opsin mutants, adeno-associated virus (AAV), cone photoreceptors, color vision deficiency

## Abstract

Long-and middle-wavelength cone photoreceptors, which are responsible for our visual acuity and color vision, comprise ~95% of our total cone population and are concentrated in the fovea of our retina. Previously, we characterized the disease mechanisms of the L/M-cone opsin missense mutations N94K, W177R, P307L, R330Q and G338E, all of which are associated with congenital blue cone monochromacy (BCM) or color-vision deficiency. Here, we used a similar viral vector-based gene delivery approach in M-opsin knockout mice to investigate the pathogenic consequences of the BCM or color-vision deficient associated L-cone opsin (OPN1LW) mutants K82E, P187S, and M273K. We investigated their subcellular localization, the pathogenic effects on cone structure, function, and cone viability. K82E mutants were detected predominately in cone outer segments, and its expression partially restored expression and correct localization of cone PDE6α’ and cone transducin γ. As a result, K82E also demonstrated the ability to mediate cone light responses. In contrast, expression of P187S was minimally detected by either western blot or by immunohistochemistry, probably due to efficient degradation of the mutant protein. M273K cone opsin appeared to be misfolded as it was primarily localized to the cone inner segment and endoplasmic reticulum. Additionally, M273K did not restore the expression of cone PDE6α’ and cone transducin γ in dorsal cone OS, presumably by its inability to bind 11-*cis* retinal. Consistent with the observed expression pattern, P187S and M273K cone opsin mutants were unable to mediate light responses. Moreover, expression of K82E, P187S, and M273K mutants reduced cone viability. Due to the distinct expression patterns and phenotypic differences of these mutants observed *in vivo*, we suggest that the pathobiological mechanisms of these mutants are distinct.

## Introduction

The genes for human long-wavelength opsin (*OPN1LW*) and middle-wavelength opsin (*OPN1MW*) are tandemly arrayed on the X-chromosome at Xq28 in a head-to-tail arrangement with a single *OPN1LW* gene in a 5′ position followed by one or more *OPN1MW* genes (Nathans et al., 1986). These genes are likely to have derived from unequal recombination and therefore share 96% identity in their DNA coding sequences. It has been shown that only the first two genes are expressed due to their proximity to the upstream locus control region (LCR), although there is conflicting evidence on the expression of “only” the first two genes in the array (Sjoberg et al., 1998). X-linked retinal diseases resulting from mutations in the *OPN1LW*/*OPN1MW* gene cluster are associated with a wide range of visual defects including red-green color vision deficiencies (MIM 303800, MIM 303900), X-linked cone dystrophy (MIM 303700), X-linked cone dysfunction (MIM 300843), and blue cone monochromacy (MIM 303700) (Nathans et al., 1989, 1993; Winderickx et al., 1992; Ayyagari et al., 1999; Jagla et al., 2002; Carroll et al., 2004, 2009; Crognale et al., 2004; Kellner et al., 2004; Neitz et al., 2004; Michaelides et al., 2005a,b; Gardner et al., 2009, 2010; Mizrahi-Meissonnier et al., 2010). Blue cone monochromacy (BCM) is caused by complete loss or severely reduced function of both L-and M-cones. Deletions in the LCR or mutations in the coding regions of both genes resulting in absence of functional L-and M-cone pigments are the genetic causes of BCM (Nathans et al., 1989, 1993; Smallwood et al., 2002; Gardner et al., 2009; Buena-Atienza et al., 2016).

Though very rare, point mutations have been identified in subjects with congenital BCM or color-vision deficiency. In a previous study, we used an AAV-based gene delivery approach to investigate the *in vivo* expression pattern and pathobiology of five cone opsin missense mutations (N94K, W177R, P307L, R330Q, and G338E) after expression in M-opsin knockout (*Opn1mw^−/−^*) mice (Zhu et al., 2021). In this study, we characterize the disease mechanisms of three additional cone opsin missense mutants that have been shown to cause color-vison deficiency or BCM (Neitz et al., 2004; Katagiri et al., 2018; Cai et al., 2019). We expressed OPN1LW cone opsin mutants K82E, P187S, and M273K specifically in *Opn1mw^−/−^* and WT cones using a cone-specific PR2.1 promoter (Mancuso et al., 2009). We then investigated the subcellular localizations of these cone opsin mutants in the *Opn1mw^−/−^* retinas, their impact on other cone outer segment (COS) proteins, and the pathogenic effects on cone structure, function, and cone viability. The results of our study will be helpful to develop future treatment strategies for color vision dystrophies.

## Materials and methods

### Animals

Mice used in this study were maintained on a 12-h light/12-h dark cycle. M-opsin knockout (*Opn1mw^−/−^*) mice have been previously described (Zhang et al., 2017). We have demonstrated previously that there are no differences in cone morphology, retinal structure, or scotopic/photopic ERG responses between *Opn1mw^−/−^* hemizygous males and homozygous females, so both were used in this study (Zhang et al., 2017; Deng et al., 2018). All experimental procedures involving animals in this study were approved and conducted in strict accordance with relevant guidelines and regulations by the Institutional Animal Care and Use Committee at University of Florida and West Virginia University, the ARVO Statement for the Use of Animals in Ophthalmic and Vision Research, and the National Institutes of Health.

### Cloning of AAV vectors expressing human OPN1LW mutants

Human *OPN1LW* missense mutations were generated using a Q5^®^ site-directed mutagenesis kit (New England Biolabs, Ipswitch, MA, USA) following a manufacturer provided protocol and a previously characterized plasmid used by the lab that contains *OPN1LW* cDNA with an HA tag fused in-frame at the C-terminus. After mutagenesis, each mutant was then cloned into an AAV vector containing a cone-specific PR2.1 promoter (Mancuso et al., 2009) and sequences were confirmed by Sanger sequencing. All mutant vectors were packaged in AAV serotype 8 Y733F by transfection of HEK293 cells and purified according to previously published methods (Zolotukhin et al., 2002).

### Subretinal injections

Before injection, eyes of 1-month-old *Opn1mw^−/−^* mice were dilated using Tropi-Phen drops (Pine Pharmaceuticals, Tonawanda, NY) approximately 15–30 min prior to administration of anesthesia by intramuscular (IM) injection of ketamine (80 mg/kg) and xylazine (10 mg/kg) in sterile phosphate buffered saline (PBS). Trans-corneal subretinal injections were then carried out using a 33-gauge blunt end needle attached to a 5 mL Hamilton syringe after an entering puncture was placed with a 25-gauge needle at the edge of the cornea. Viral vector mixed with fluorescein dye (0.1% final concentration, 1 μL volume of mixture) was delivered into the subretinal space as described previously (Pang et al., 2006, 2008). Eyes were treated post-injection with Neomycin/Polymixin B Sulfates/Bacitracin Zinc ophthalmic ointment (Bausch & Lomb, Inc., Tampa, FL). An intraperitoneal (IP) injection of antisedan (Orion Corporation, Espoo, Finland) was then given to reverse anesthesia.

### Immunoblot analysis

Following humane euthanasia of mice, retinas from uninjected and mutant opsin injected *Opn1mw^−/−^* mice, as well as from wild-type (WT) controls (three of each), were carefully dissected and flash frozen on dry ice. Each retinal sample was then homogenized by sonication in 300 μL of protein extraction buffer [0.23 M sucrose, 2 mM EDTA, 5 mM Tris–HCl (pH 7.5)] containing 1X cOmplete^™^ protease inhibitor cocktail (Millipore Sigma, Burlington, MA, USA). Supernatant was collected after centrifugation and a Pierce^™^ BCA Protein Assay Kit was used to determine protein concentration. Retinal lysate was then divided into aliquots containing equal amounts of total protein (40 μg) and subjected to SDS-PAGE gel electrophoresis on a 4–15% Mini-PROTEAN^®^ TGX^™^ Precast Gels (Bio-Rad, Hercules, CA, USA). After SDS-PAGE gel electrophoresis, proteins were transferred to an Immobilon-FL membrane (Millipore Sigma, Burlington, MA, USA) and probed with anti-red/green opsin (Millipore, AB5405, 1:1000 dilution) antibody. Anti-alpha tubulin (cat # T5168, 1:5000; Millipore Sigma, Burlington, MA, USA) served as a loading control. Immunoblot was visualized with the Odyssey Infrared Fluorescence Imaging System (LICOR Biosciences, Lincoln, NE, USA).

### Electroretinography

Preceding ERG testing, animals were light-adapted to inhibit rod responses and pupils were dilated with Tropi-Phen drops (Pine Pharmaceuticals, Tonawanda, NY). Photopic ERGs were carried out 4-weeks post-injection using a UTAS Visual Diagnostic System with a Big Shot Ganzfeld dome (LKC Technologies, Gaithersburg, MD, USA). For ERG testing, all mice were anesthetized with ketamine (80 mg/kg) and xylazine (10 mg/kg) by IM injection. Cone ERGs from mutant opsin injected eyes were recorded with green channel middle wavelength light (530 nm) stimulation followed by red channel long wavelength light (630 nm), both at various intensities (−0.6, 0.4, and 1.4 log cd s/m^2^). Both M-cone (green) and L-cone (red) ERGs produced similar ERG results. Red light ERG recordings taken at 1.4 log cd s/m^2^ were used for statistical analysis and for plotting data. S-cone ERGs (360 nm) were recorded at intensities of −0.6 and 0.4 log cd s/m^2^. ERG data are presented as average ± SD (*n* = 8 for each mutant injected group). Flat ERGs lacking a measurable a-or b-wave were assigned a b-wave maximum amplitude of 1 μV. Statistical analysis of ERG data comparing mutant injected eyes vs. untreated and WT controls is performed by one-way ANOVA and Dunnett’s multiple comparisons test (GraphPad Prism). Statistical significance is defined as a *p* value of <0.01.

### Frozen retinal section preparation and immunohistochemistry

After euthanasia, eyes were marked at the dorsal position using the Change-a-Tip Deluxe Cautery tool (Braintree Scientific, Braintree, MA). Next, eyes were enucleated and a 16-gauge needle was used to introduce an opening along the edge of the cornea before being incubated in 4% paraformaldehyde (PFA) in 1X PBS for 2 h at room temperature. The cornea was dissected carefully away from the eye and the lens was removed. Next, eyecups were rinsed in 1X PBS and placed in 30% sucrose overnight at 4°C for cryoprotection before being embedded in Tissue TEK O.C.T. compound (Sakura Finetek USA, Inc., Torrance, CA) and frozen at −80°C. Retinas were sectioned from dorsal to ventral at 12 μm thickness. For immunohistochemistry (IHC), retinal cross-sections were rinsed in 1X PBS and then blocked [3% Bovine serum albumin (BSA), 0.3% Triton X-100 in 1X PBS] for 1 h at room temperature before incubating in primary antibody overnight at 4°C. Anti-L/M-opsin (MilliporeSigma, AB5405, 1:1000), anti-S-opsin (MilliporeSigma AB5407, 1:1000 dilution), anti-HA (Roche, rat monoclonal, clone 3F10, 1:200), anti-PDE6α’ (ABgene, AP9728c, 1:200), and anti-cone transducin γ subunit (gift from Dr. Vadim Arshavsky’s lab, Duke University) antibodies were used. Following primary incubation, sections were washed and incubated with Alexa-594 or Alexa-488 IgG secondary antibodies (molecular Probes, Eugene OR, 1:500) at room temperature for 2 h. Sections were washed after secondary and subsequently mounted with Vectashield Mounting Medium for Fluorescence (H-1400, Vector lab, Inc. Burlingame, CA, USA) and cover slipped. A Leica Fluorescence Microscope LAS X Widefield System was used to image the stained sections.

### Preparation of retinal whole mounts and PNA staining

Prior to enucleation, a Change-a-Tip Deluxe Cautery tool (Braintree Scientific, Braintree, MA) was used to mark eyes on the cornea at the dorsal position. A 16-gauge needle was used to poke a hole along the edge of the cornea before the enucleated eye was introduced into 4% PFA in 1X PBS. The cornea and lens were removed following a 2-h incubation at room temperature and a radial cut was done to mark the dorsal position. After dissecting the neural retina from the eyecup, retinal whole mounts were blocked in 3% BSA containing 0.3% Triton-X-100 in 1X PBS overnight at 4°C. On the next day, retinal whole mounts were placed in primary antibody containing biotinylated peanut agglutinin (PNA; Vector Laboratories, Burlingame, CA, 1:500) and anti-S-opsin antibody in 1% BSA in 1X PBS overnight at 4°C. Whole mounts were subsequently washed in 0.05% Tween-20 in 1X PBS before being incubated in secondary antibody containing Fluorescein Avidin D (Vector Laboratories, Burlingame, CA, 1:500) and anti-rabbit IgG Alexa-594 (1:500) in 1X PBS. Finally, whole mount retinas were washed and ventral, temporal, and nasal cuts were made before being coverslipped using ProLong Gold Antifade Mountant (Thermo Fisher, Waltham, MA). Whole mounts were imaged using a Nikon C2 confocal microscope and processed using FIJI software (Schindelin et al., 2012). Four images were captured (two ventral, two dorsal) for each retinal whole mount, and PNA-positive cells from K82E (*n* = 6 eyes), P187S (*n* = 8), and M273K (*n* = 6) images were counted within an area equivalent of 0.04 mm^2^ per image using the particle analysis tool in ImageJ. Counts were averaged for the dorsal and ventral regions and the SD was calculated. Statistical analysis was performed by two-way ANOVA with Tukey’s multiple comparisons test to compare differences between each mutant group for dorsal or ventral regions (*p ≤ 0.05, **p < 0.002, ***p < 0.001).

### Statistical analysis

All data are presented as the mean ± SEM unless otherwise noted. Figure legends contain details about sample size. All analyses were carried out using GraphPad Prism 9 software by ordinary 1-way ANOVA with Dunnett *post hoc* test (comparing two groups) or by two-way ANOVA with Dunnett *post hoc* test for multiple comparisons unless noted differently. Significance is indicated as *p ≤ 0.05, **p < 0.002, or ***p < 0.001.

## Results

### Expression of cone opsin mutants *in vivo*

**Figure 1:**
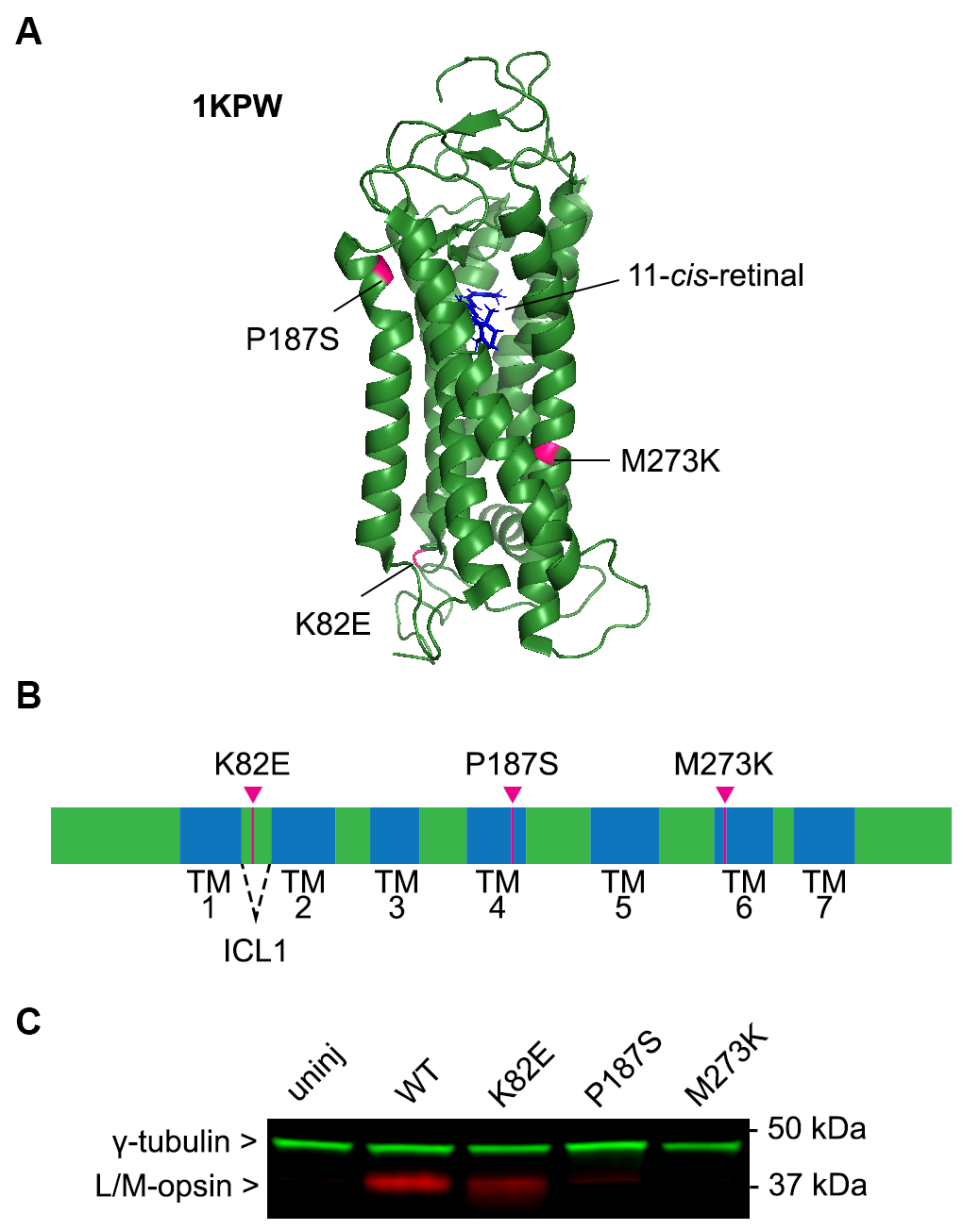
Expression and function of cone opsin mutants. **(A)** Mutation locations labeled on the predicted cartoon structure of green cone pigment (OPN1MW, PDB: 1KPW; drawn with PyMOL, Molecular Graphics System, Version 1.2r3pre, Schrödinger, LLC). **(B)** Linear schematic of human green opsin depicting the location of these mutant residues. K82E is located within intercellular loop 1 (ICL1), P187S in transmembrane (TM) helix 4, and M273K in TM6. **(C)** Immunoblot blot of AAV-mediated expression of cone opsin mutants in *Opn1mw^−/−^* mice 1-month post-injection. Membranes were incubated with anti-L/M-opsin (red) and anti-γ-tubulin (loading control, green) antibodies. Retinas from C57BL/6J (WT) and *Opn1mw^−/−^* (uninjected) mice were used as positive and negative controls, respectively.

The cDNA of each *OPN1LW* cone opsin missense mutant (K82E, P187S, and M273K) fused with a C-terminal HA tag was cloned into an AAV vector under a cone-specific PR2.1 promoter and packaged into an AAV8-Y733F capsid. The amino acid position of these mutations in human cone opsin are illustrated in Figures 1A,B (Stenkamp et al., 2002). Each *OPN1LW* mutant AAV vector was delivered by subretinal injection into one eye of *Opn1mw^−/−^* mice at 1 month of age, with the contralateral eye serving as an uninjected control. Retinas were collected 30 days post-injection and OPN1LW protein levels were analyzed by western blot using an anti-red/green opsin antibody that binds the C-terminus of human L/M-opsin (Jones et al., 2011). We were able to identify OPN1LW-K82E expression in injected eyes. However, expression of OPN1LW-P187S was minimal and we were unable to detect expression of OPN1LW-M273K (Figure 1C).

### Light responses of missense cone opsin mutants

**Figure 2:**
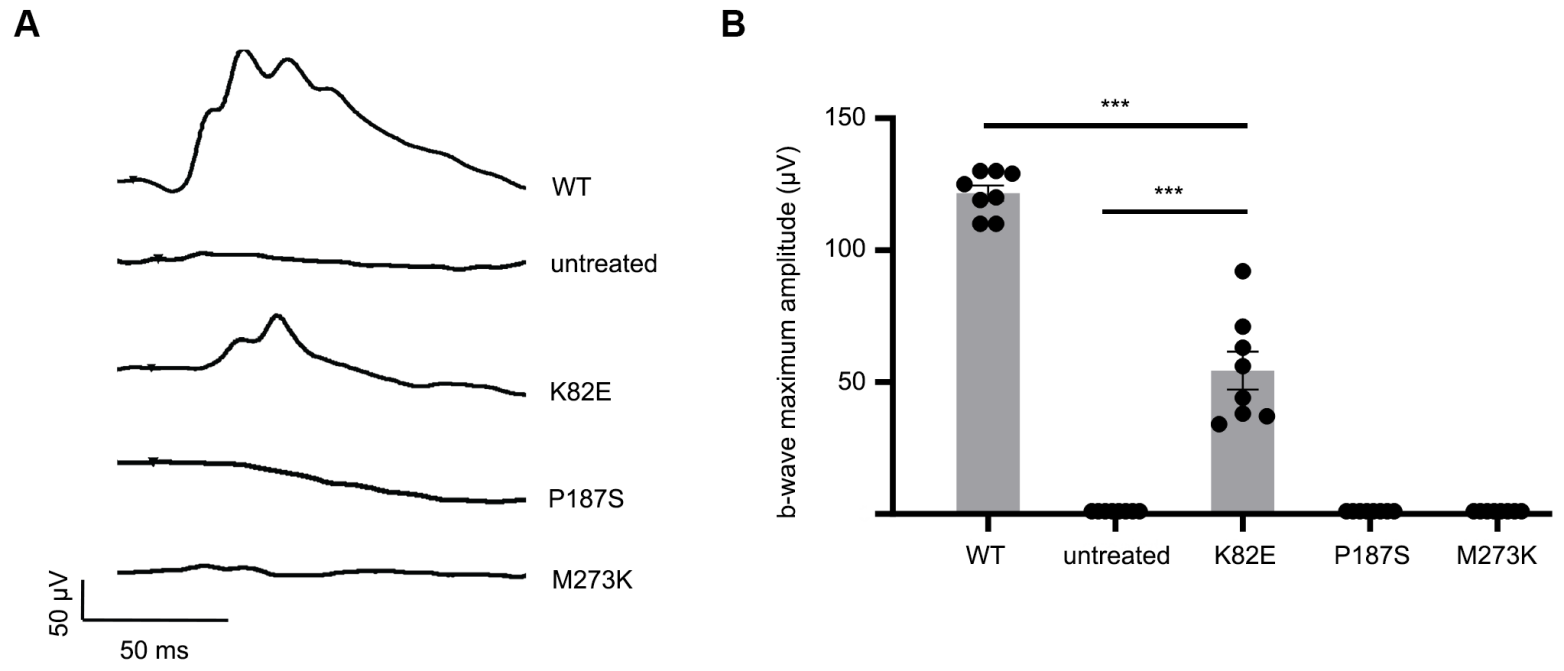
Cone opsin mutants K82E, P187S, and M273K demonstrate severe reduction or inability to mediate light responses. **(A)** Representative ERG traces of K82E, P187S, and M273K injected *Opn1mw^−/−^* eyes at 1.4 log cd●s/m^2^ under 630 nm wavelength light. **(B)** Individual and averaged ERG responses from each mutant opsin injected *Opn1mw^−/−^* eye. Data presented as the mean ± SEM of b-wave maximum amplitudes recorded at 1.4 log cd●s/m^2^ (*n* = 8 for each group). One-way ANOVA used for statistical analysis (***p < 0.001). Untreated *Opn1mw^−/−^* and wild type mice served as controls.

To determine the capability of these OPN1LW missense mutants in generating responses to light, we performed long- (630 nm) wavelength cone ERGs on treated *Opn1mw^−/−^* mice at 1-month post-injection. Uninjected *Opn1mw^−/−^* eyes do not exhibit an L-or M-cone mediated light response but have a normal S-cone ERG (Zhang et al., 2017). We were unable to detect green-or red-light ERG responses in *Opn1mw^−/−^* eyes expressing mutant P187S or M273K. However, K82E-injected eyes generated an average b-wave amplitude of 54.4 ± 20.2 μV (average ± SD, *n* = 8), demonstrating a visual response that is significantly lower than WT controls (121.6 ± 8.3 μV, *n* = 8, *p* < 0.0001), but significantly higher than the flat ERGs from untreated contralateral control eyes (*p* < 0.0001) (Figures 2A,B). Together, these results suggest that the only OPN1LW mutant in this study that is capable of generating responses to light is K82E, but its efficiency to do so is reduced in comparison to WT OPN1LW.

### Subcellular localizations of OPN1LW mutants

We next determined the subcellular localization of these OPN1LW mutants in injected *Opn1mw^−/−^* eyes by immunohistochemistry (IHC). Previously, we have demonstrated dorsal *Opn1mw^−/−^* cones exhibit shortened outer segments, but remain viable, while ventral cones retain normal cone structure due to the presence of S-opsin (Deng et al., 2018). In addition to each mutant, we also examined the expression and localization of two endogenous COS-specific proteins involved in phototransduction, phosphodiesterase α subunit (PDE6α’) and cone transducin γ subunit (GNGT2), in both the dorsal and ventral regions of the injected *Opn1mw^−/−^* retinas.

**Figure 3:**
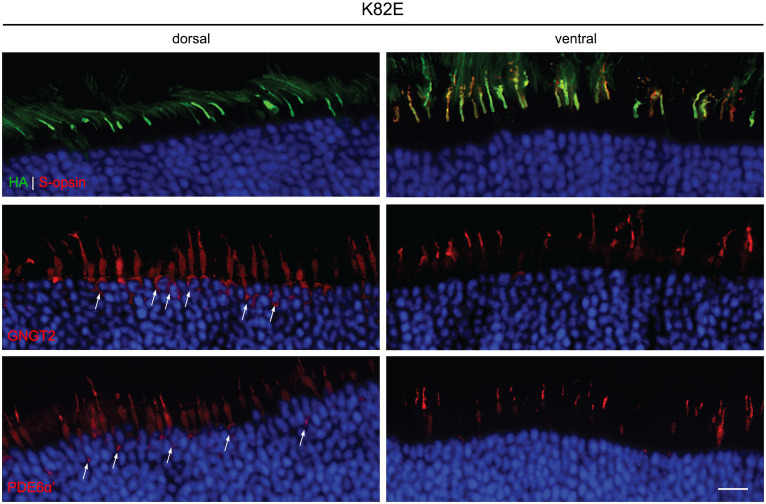
Subcellular localization of HA-tagged K82E opsin, GNGT2, and PDE6α’ in *Opn1mw^−/−^* injected retinas. K82E (HA, green, top panels) was expressed within the cone outer segment in both dorsal and ventral retinas, co-localizing with endogenous S-opsin (red) in the ventral region. GNGT2 (middle panels) and PDE6α’ (bottom panels) were observed in cone outer segments, inner segments, and in the ER (arrows) within the dorsal retina, but localized normally to the cone outer segments in the ventral area of the same eye. Scale bar: 20 μm.

Localization of K82E was detected mainly within the COS, both in the dorsal and ventral retina (Figure 3). GNGT2 and PDE6α’ were localized normally to the COS within the ventral retinas that express S-opsin. However, in the dorsal retina, both proteins were partially mislocalized to the inner segment and endoplasmic reticulum (ER). Previous studies have demonstrated that binding of 11-*cis*-retinal is required for proper trafficking of cone opsins to the COS (Zhang et al., 2008). Furthermore, trafficking of downstream phototransduction proteins cone transducin and cone PDE6 co-traffic with 11-*cis*-retinal bound cone opsin. Therefore, these results suggest that while K82E mutants properly bind chromophore and are correctly transported to the COS, binding and trafficking of GNGT2 and PDE6α’ to the OPN1LW mutant is inhibited as these proteins are trapped in the inner segment. The mislocalization of these two proteins explains the partial cone ERG rescue observed in K82E-injected eyes, as the cones lack the proper levels of phototransduction proteins in the light-sensing outer segments.

**Figure 4:**
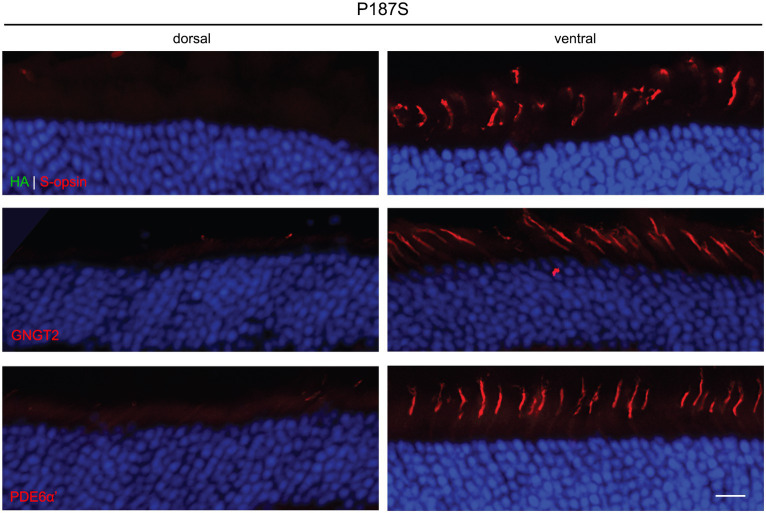
Subcellular localization of HA-tagged P187S opsin, GNGT2, and PDE6α’ in *Opn1mw^−/−^* injected retinas. We were unable to detect P187S expression (HA, green, top panels) in either the dorsal or ventral region of injected retinas, but observed endogenous S-opsin (red) in the ventral retinas. GNGT2 (middle panels) and PDE6α’ (bottom panels) expression were also not detected in the dorsal retina, but were expressed normally in the ventral retina to the cone outer segments. Scale bar: 20 μm.

**Figure 5:**
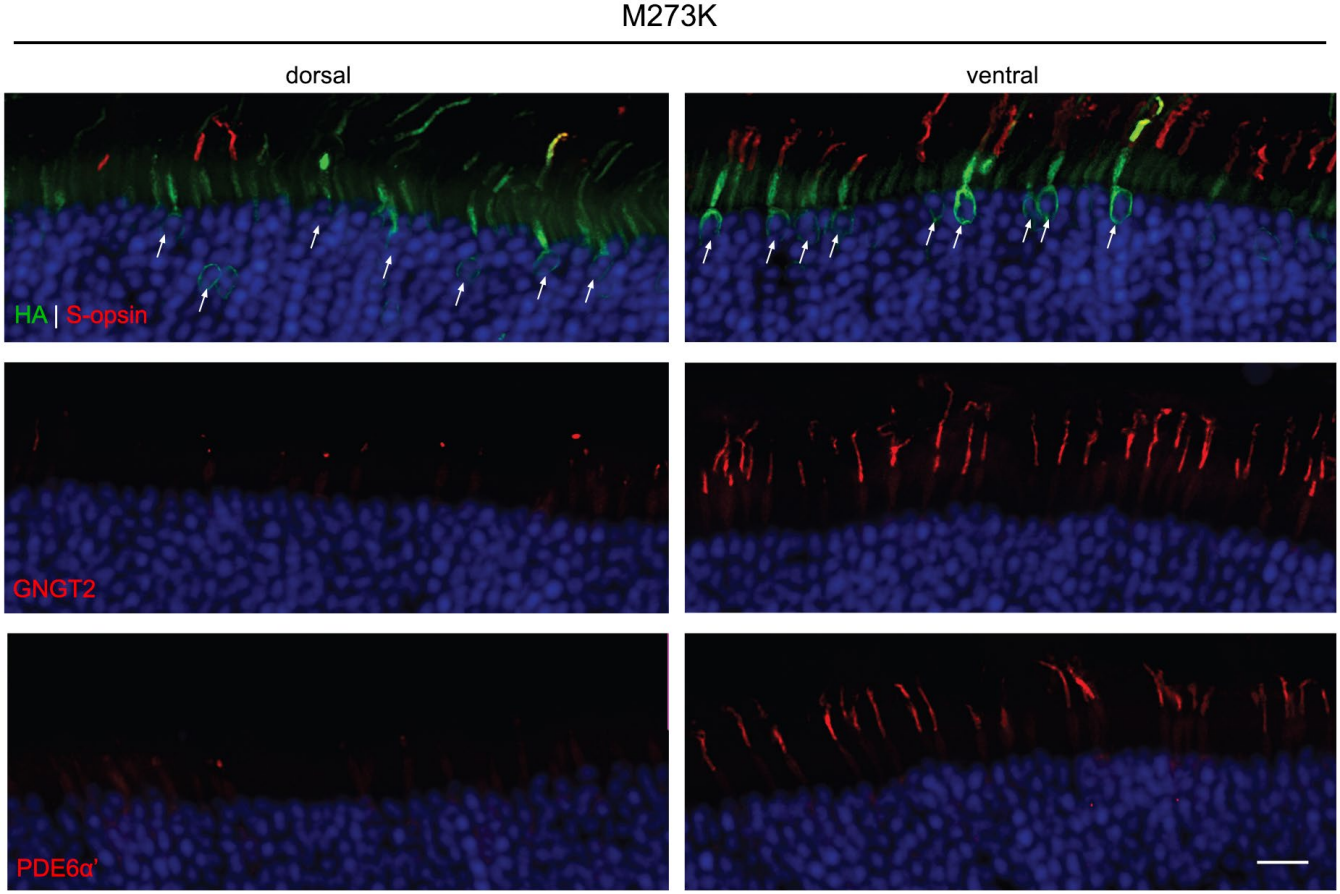
Subcellular localization of HA-tagged M273K opsin, GNGT2, and PDE6α’ in *Opn1mw^−/−^* injected retinas. M273K (HA, green, top panels) opsin appeared to be misfolded and localized exclusively to the cone inner segment and ER in both the dorsal and ventral retinas. GNGT2 (middle panels) was barely detectible in the dorsal area while it was expressed normally and localized to the cone outer segment in the ventral area of the same eye where S-opsin was present. PDE6α’ (bottom panels) was undetectable in the dorsal retina but was localized and expressed normally to cone outer segments in the ventral retina. Scale bar: 20 μm.

We were unable to detect expression of P187S by IHC in both the dorsal and ventral region of injected retinas. In the dorsal region that lacks endogenous M-opsin or AAV-mediated P187S, GNGT2 and PDE6α’ were either undetectable or severely reduced, but appeared to be localized normally in the ventral retina where S-opsin is present (Figure 4). These data suggest that P187S is misfolded and fails to exit the perinuclear ER, preventing co-trafficking of GNGT2 and PDE6α’. Interestingly, while we were unable to detect M273K by western blot analysis, IHC staining showed that M273K was localized to the cone inner segment and ER in both the dorsal and ventral areas of injected *Opn1mw^−/−^* retinas, suggesting that M273K is likely partially misfolded, which may interfere with efficient 11-*cis*-retinal binding (Figure 5). PDE6α’ and GNGT2 were barely detectible in the dorsal retinas, but were expressed normally and localized correctly to the COS in cones expressing S-opsin within the ventral region of the same retina.

### Cone viability of WT eyes expressing different OPN1LW mutants

**Figure 6:**
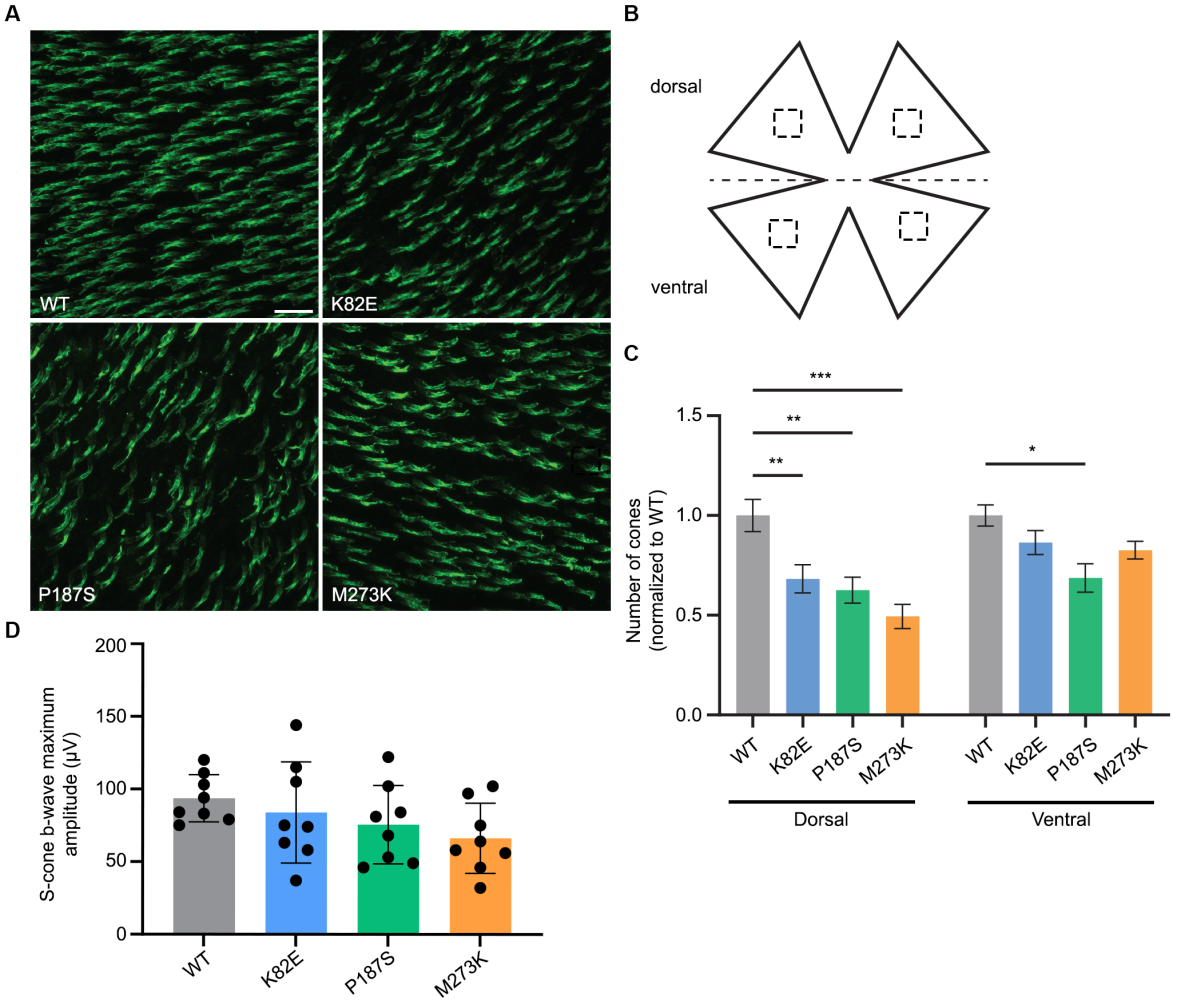
Cone viability and S-cone function in WT retinas injected with cone opsin mutants. **(A)** Representative images of WT retinal flatmounts injected with K82E, P187S, or M273K cone opsin mutants at 1-month post-injection. Flatmounts were stained with PNA to show cone viability. Scale bar = 20 μm. **(B)** Cone density was calculated by counting the number of PNA^+^ cells in four regions (two dorsal, two ventral) from retinal flatmounts, as indicated by the dashed squares. **(C)** Quantification of cone density from the dorsal (left) and ventral (right) regions of K82E (*n* = 6 eyes), P187S (*n* = 8), and M273K (*n* = 6) injected WT retinal flatmounts. Uninjected WT eyes served as controls. Each bar represents the number of PNA^+^ cones normalized to WT. Data is shown as the average ± SEM, two-way ANOVA was used for statistical analysis (*p ≤ 0.05, **p < 0.002, ***p < 0.001). **(D)** Averaged S-cone ERG responses from each mutant opsin injected WT eye. Data presented as the mean ± SD of b-wave maximum amplitudes recorded at −0.6 log cd●s/m2 under 360 nm wavelength light (*n* = 8 for each group). Untreated WT mice served as controls. One-way ANOVA revealed that K82E (*p* = 0.86), P187S (*p* = 0.39), or M273K (*p* = 0.08) cone opsin mutant injected eyes did not demonstrate statistically significant reductions in S-cone function.

To determine if expression of the *OPN1LW* mutants has a toxic effect on cone viability, we next examined the retinas of WT eyes injected with each mutant. Cone viability was assessed by peanut agglutinin (PNA) staining by counting the number of PNA^+^ cells in both the dorsal and ventral retina and compared to the contralateral, uninjected eyes at 1-month post-injection (Figures 6A–C). PNA specifically binds to the extracellular glycoprotein matrix of cone outer and inner segment sheaths and is an indicator of cone viability (Blanks and Johnson, 1983). All three mutants significantly reduced the number of viable cones in the dorsal retina compared to untreated WT controls to a various extent, ranging from a 31% to a 74% decrease, but only P187S (31.3% decrease) also showed a significant reduction in viable cones within the ventral retina (Figure 6C). Short- (360 nm) wavelength ERGs were also performed on WT injected eyes to determine the toxicity of these various L-opsin mutants on S-cone function. Measurements of S-cone ERGs in each mutant treated eye showed that expression of K82E, P187S, or M273K did not result in a significant ERG reduction compared to WT eyes (Figure 6D), suggesting these mutant proteins do not alter cone viability but are partially or severely misfolded.

## Discussion

In the current study, we characterized three known patient missense mutations in cone opsin that are linked to BCM or color vision deficiency. We employed a viral vector-based approach to delivery these mutants in mice lacking M-opsin (*Opn1mw^−/−^*) in order to investigate the subcellular localization, as well as the pathogenic effects these mutants have on cone structure, function and viability. Our results demonstrate that each OPN1LW cone opsin mutant displays a distinct expression pattern and phenotypic consequence on the function and structure of injected cones, suggesting that the pathobiological mechanisms of these mutants are different. These results provide basic knowledge and foundation for delineating effective treatment strategies.

Lysine 82 is located within the intracellular loop 1 (ICL1) of red and green cone opsin. Studies have shown that several positively charged residues in the ICL1 and ICL3 of rhodopsin form electrostatic interactions with phosphorylation sites near its C-terminus, including Lysine 67 within ICL1 (Jatana et al., 2020). As rhodopsin phosphorylation bolsters high-affinity binding of arrestin in order to shutoff phototransduction, it is possible that disruption of this electrostatic interaction with a positively charged residue in ICL1 would alter its conformation (Wilden et al., 1986; Pitcher et al., 1998). Therefore, substitution of the positively charged lysine residue for a negatively charged glutamic acid at amino acid position 82 could explain the observed decrease in the ability for K82E cone opsin to mediate an efficient light response if arrestin binding is disrupted, however, further work must be carried out to confirm this possibility. Based on studies which demonstrate that chromophore binding is crucial in proper trafficking of cone opsin to the COS (Zhang et al., 2008) and that cone transducin/cone PDE6 bind cone opsin for co-trafficking, our findings suggest that K82E does bind 11-*cis*-retinal, as it properly trafficked to the COS. However, it appears that binding and co-transport of GNGT2 and PDE6α’ are likely affected, as these downstream phototransduction proteins were mainly localized to the inner segment and within the ER. As K82E mutant opsin appears to be stably expressed, presence of this mutant in the COS requires consideration when designing gene therapy strategies to improve vision in corresponding patients, as our findings suggest this missense mutation may contribute to a dominant negative disease mechanism.

Proline 187 is conserved across the vertebrate visual pigments in the G-protein coupled receptor (GPCR) (Hwa et al., 2001). Based on crystal structure of rhodopsin, this proline generates a significant bend at the extracellular end of the fourth transmembrane alpha helical segment (Palczewski et al., 2000). Mutation of this conserved proline residue likely disrupts the proper folding and binding of 11-*cis*-retinal. Importantly, several patient mutations in the corresponding proline in rhodopsin have been identified and linked to autosomal dominant retinitis pigmentosa (adRP), including P171S, P171Q, and P171L (Dryja et al., 1991; Antiolo et al., 1994; Vaithinathan et al., 1994; Zhen et al., 2023). The fact that we were unable to detect expression of P187S mutant opsin by IHC and only slightly by western blot further supports this notion that this mutant protein is likely degraded due to protein misfolding.

Methionine 273 is located in transmembrane helix VI and is conserved between visual GPCRs. Previous *in vitro* studies in HEK293 cells have shown that recombinant M273K mutant protein displays no absorbance at any tested wavelength, even after reconstitution with 11-*cis*-retinal, likely due to its inability to bind 11-*cis*-retinal (Katagiri et al., 2018). Here, we show that M273K mutant opsin expressed *in vivo* is mislocalized to the ER and cone inner segment. Interestingly, we were unable to detect M237K protein by western blot, likely due to aggregation of mutant protein in the ER. Consistent with the previous *in vitro* study, M273K-injected eyes were unable to generate a response to light. Together, these results demonstrate that an M273K mutation results in misfolding and dysfunction of the mutant opsin protein, likely due to impeded 11-*cis*-retinal chromophore binding.

To date, our lab has characterized two mouse models which phenocopy the two most common causes of BCM, *Opn1mw^−/−^Opn1sw^−/−^* mice for BCM with deletions and *Opn1mw^C198R^Opn1sw^−/−^* mice for the corresponding human C203R missense mutation (Ma et al., 2022; Sechrest et al., 2023, 2024). We showed in both models that AAV-mediated gene replacement or augmentation of human L-or M-opsin rescues cone function, initiates extension of the COS, and results in replenishment of crucial COS phototransduction proteins. However, cones in these two models lack expression of functional opsin due to deletion or degradation of the misfolded C203R opsin, allowing for exclusive expression of AAV-mediated normal M-or L-opsin. This method of gene replacement therapy would possibly work for P187S where mutant cone opsin protein is barely detectible, but would likely not be efficacious when a dominant negative mutant cone opsin such as K82E or M273K is expressed. As stated previously in our studies of other cone opsin missense mutants which are abundantly expressed, including N94K, P307L, R330Q, and G338E, it will be important to consider the possible toxic dominant negative effect or gain-of-function of mutant cone opsin when normal opsin is supplemented. Therefore, a knockdown (shRNA) to remove the toxic mutant opsin in tandem with expression of a “hardened” (shRNA resistant) normal cDNA, or CRISPR/Cas9 gene editing approach might be needed to obtain an effective therapy in these cases.

In summary, the current study is a continuation of our previous work to characterize the pathobiology of several known patient missense mutations in red and green cone opsin linked to BCM and color-vision deficiency, in order to broaden our understanding of each mutant’s underlying disease mechanism. As a result, we have identified additional dominant negative missense mutations in red and green cone opsin. These findings are useful when considering the design of treatment strategies to restore vision in patients with this currently untreatable visual disorder.

## Data availability statement

The raw data supporting the conclusions of this article will be made available by the authors, without undue reservation.

## Ethics statement

The animal study was approved by Institutional Animal Care and Use Committee at University of Florida Institutional Animal Care and Use Committee at West Virginia University. The study was conducted in accordance with the local legislation and institutional requirements.

## Author contributions

ES: Writing – original draft, Writing – review & editing, Conceptualization, Data curation, Formal analysis, Investigation, Methodology, Supervision, Validation. RB: Writing – review & editing, Investigation. XM: Writing – review & editing, Investigation. FD: Writing – review & editing, Investigation. JA: Writing – review & editing, Investigation. BB: Writing – review & editing, Investigation. MC: Writing – review & editing, Investigation. IH: Writing – review & editing, Investigation. WB: Writing – review & editing, Funding acquisition, Resources. W-TD: Writing – original draft, Writing – review & editing, Conceptualization, Data curation, Formal analysis, Funding acquisition, Investigation, Methodology, Project administration, Resources, Supervision, Validation, Visualization.
